# Benign or by Chance: A Case Report on Incidental Appendectomy Revealing a Neuroendocrine Tumor During Traumatic Exploratory Laparotomy

**DOI:** 10.7759/cureus.54527

**Published:** 2024-02-20

**Authors:** Megan B Douglass, Sheldon L Carpenter, Cayla H Campbell, Chase Hoffman, Jacob Hessey

**Affiliations:** 1 Surgery, University of South Carolina School of Medicine, Columbia, USA; 2 General Surgery, Prisma Health, Columbia, USA; 3 Critical Care, Prisma Health, Columbia, USA

**Keywords:** exploratory laparotomy, peritonitis, incidental appendectomy, loop colostomy, appendiceal neuroendocrine tumor

## Abstract

Incidental appendectomies (IAs) are often performed in laparotomies to prevent future complications caused by the buildup of scar tissue. Although neoplastic findings are rare, all appendectomy specimens should be sent for histopathological analysis. We present the case of a 38-year-old man found to have an appendiceal neuroendocrine tumor (NET) after receiving an IA secondary to a traumatic rectal perforation requiring exploratory laparotomy. Well-differentiated NETs isolated to the appendix have an excellent prognosis. Appendectomies are considered curative for NETs smaller than 2 cm that have not metastasized beyond the appendix. Appendiceal NETs are capable of secreting vasoactive substances and, therefore, causing carcinoid syndrome. However, the progression to carcinoid syndrome generally coincides with metastasis to the liver, indicating a poor prognosis. While histopathological analysis of appendectomy specimens rarely yields atypical findings, this analysis is crucial to ensure that the proper treatment is selected based on tumor progression in an appendectomy specimen staining positive for somatotropin and chromogranin.

## Introduction

Incidental appendectomies (IAs) may be performed on patients undergoing laparotomy due to the potential complications that scar tissue can present in a future case of appendicitis [1]. IAs have a low complication rate, making them a viable option in clinical scenarios requiring laparotomy wherein a risk-to-benefit analysis for the particular patient is performed [1]. While only about 1% of appendectomy specimens are found to be cancerous [2], it is generally considered the standard of care for all appendectomy specimens to be sent for histopathological analysis [3]. 

Preoperative imaging, including computed tomography (CT), magnetic resonance imaging (MRI), and ultrasound, can be used to detect larger neuroendocrine tumors (NETs) and potential metastasis [4]. 

A rectal perforation can be a severe injury requiring immediate medical attention. Some of the complications of rectal perforation include bleeding, peritonitis, and sepsis [5]. Different options are available for repairing rectal perforations, depending on the size and location of the perforation, as well as the patient's overall health. Smaller perforations may be treated conservatively with close monitoring and antibiotics, while most perforations require surgery for repair [6].

There are different surgical options available depending on the severity and location of the perforation, including endoscopic repair, laparoscopic surgery, and exploratory laparotomy [6]. Exploratory laparotomy, although it carries the most significant risk of post-surgical complications such as infection, is utilized in emergency situations where the patient is showing signs of sepsis or peritonitis, as was seen in this particular case. Exploratory laparotomies carry mortality rates between 13% and 18%, which is five times greater than the mortality rate for high-risk elective surgeries [7]. Some of the most common complications from an exploratory laparotomy include surgical site infection and scar tissue formation [7].

## Case presentation

A 38-year-old male presented to the emergency department with rectal bleeding and abdominal pain for three hours after receptive anal intercourse. The patient was previously healthy with no significant past medical history. His past surgical history included a laparoscopic cholecystectomy. Upon physical examination, the patient was ill-appearing, and his abdomen was diffusely rigid with peritoneal signs and guarding present. The patient's vital signs were significant for hypertension (149/92). All other vital signs were within normal limits. The patient's complete blood count (CBC) was notable for an elevated white blood cell count of 30,900 per microliter. His Mannheim Peritonitis Index for Mortality Prediction resulted in a score of 22, indicating a mortality rate of 26% [8].

**Figure 1:**
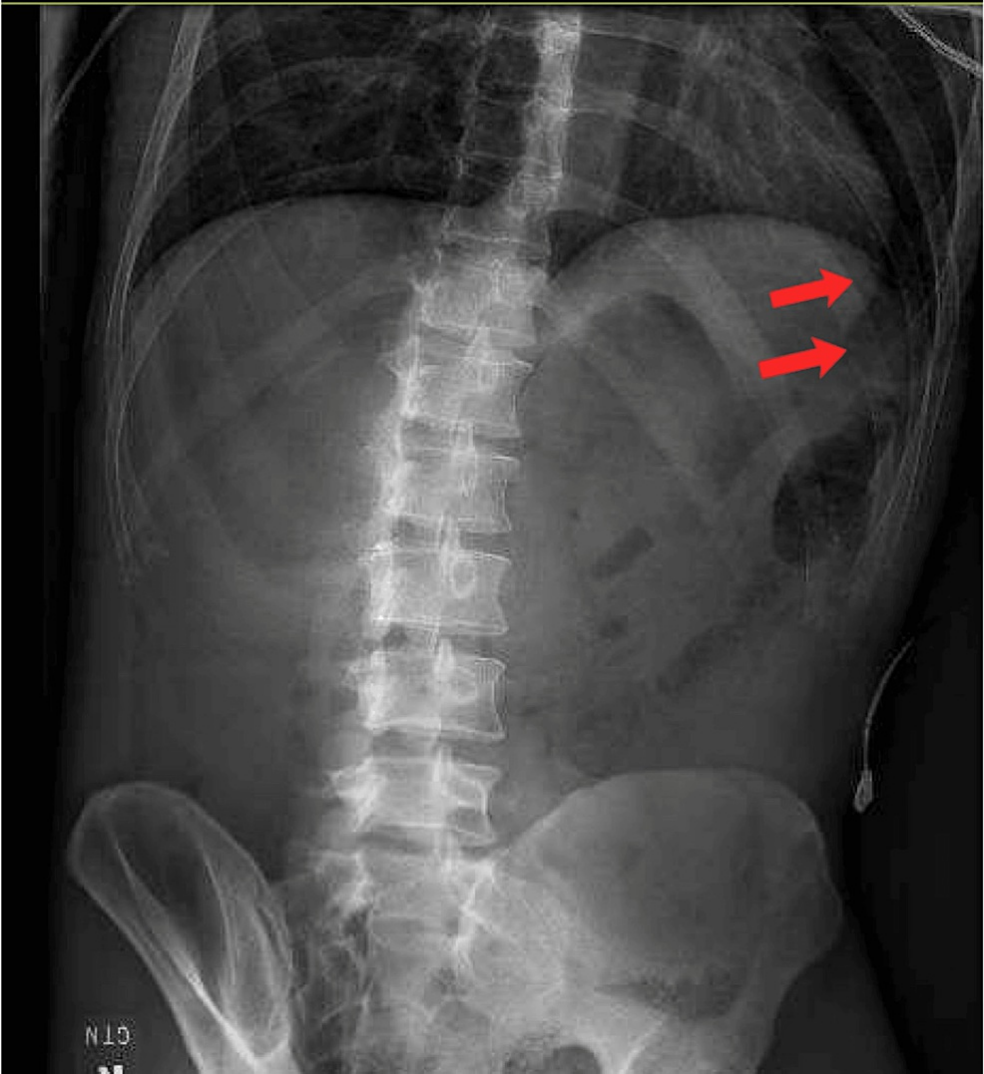
Abdominal X-ray indicating potential extraluminal gas The red arrows indicate an area of lucency in the upper abdomen, raising suspicion for extraluminal gas.

Given the patient's presentation and physical examination, rectal perforation was highly suspected. An abdominal X-ray (Figure 1) was immediately ordered, indicating a concern for intra-abdominal extraluminal air. General surgery was consulted secondary to this finding.

**Figure 2:**
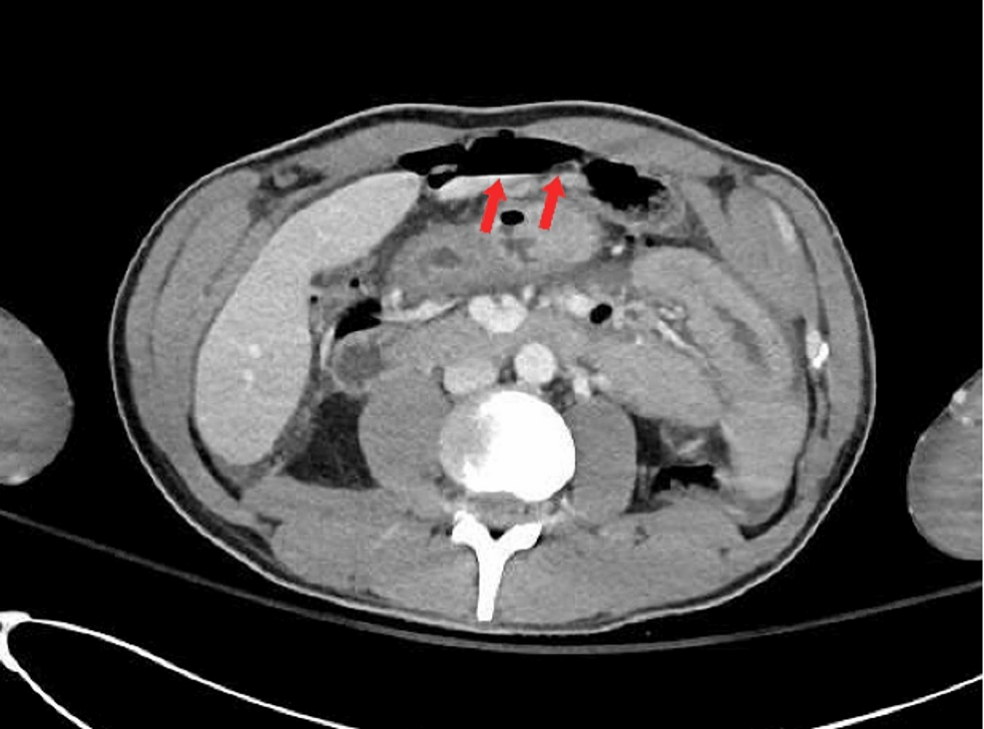
Abdominal CT scan exhibits extraluminal gas representing perforated viscus The red arrows in the figure point out the extraluminal gas, which appears black. CT: computed tomography

An abdominal CT scan was recommended to confirm the abdominal X-ray findings. The patient received two large IVs for fluid resuscitation and antibiotic therapy (ceftriaxone and metronidazole) as a preventive measure. Given the patient's stable vital signs, he was taken for an abdominal CT scan with IV contrast (Figure 2), which indicated pneumoperitoneum with discontinuation in the rectal wall. 

**Figure 3:**
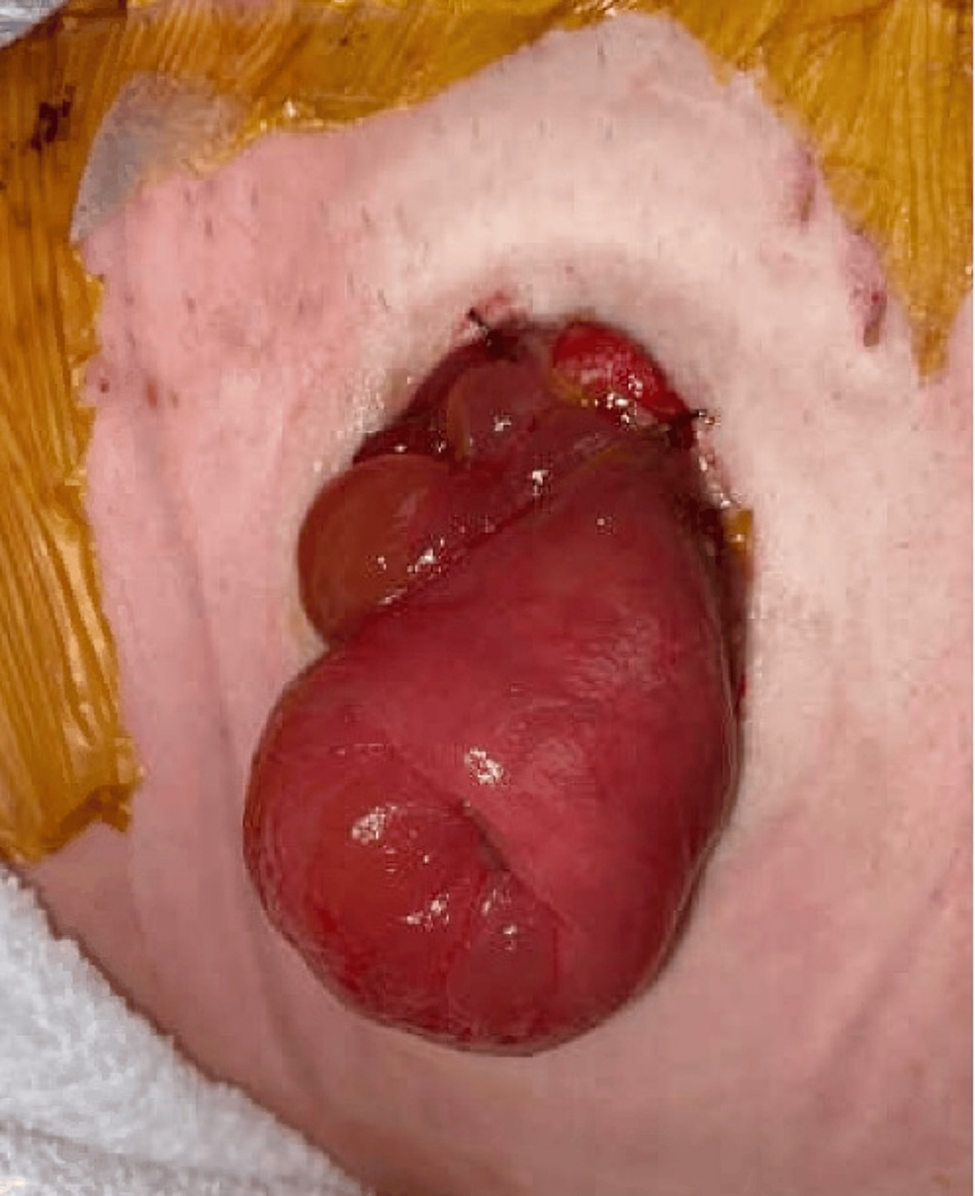
Loop sigmoid colostomy for stool diversion

Upon these findings, the patient was taken immediately to the operating room for an exploratory laparotomy and possible bowel resection and colostomy creation. Before the operation, the patient received IV ceftriaxone and metronidazole. A midline incision was made from the umbilicus to the pubic tubercle. A Bookwalter retractor (Symmetry Surgical, Nashville, Tennessee, United States) was used to visualize the abdomen, revealing feculent peritonitis. The abdomen was washed out using 3 L of normal saline. The perforation, comprising approximately half of the bowel circumference, was visualized in the anterior portion of the upper rectum. The primary rectal injury, measuring approximately 3 cm, was repaired transversely using interrupted 3-0 polydioxanone (PDS) sutures, with several Lembert 2-0 silk sutures being placed over this repair. It was not necessary to trim the bowel before placing the sutures, as none of the bowel was necrotic. A loop sigmoid colostomy for stool diversion was conducted to allow for adequate wound healing. A circular incision was made in the patient's left lower quadrant, with the sigmoid colon pulled through (Figure 3).

Due to several variables relating to this patient's presentation, the decision was made to conduct an appendectomy during the exploratory laparotomy. The patient was hemodynamically stable and did not require the use of pressors. Also, given the existing intraperitoneal contamination, the additional risk posed by appendectomy was low. Furthermore, the scar tissue from this procedure would make a future appendectomy difficult for this patient, should it be needed. The appendectomy was sent for pathological analysis. The abdominal cavity was then washed out with a clindamycin and gentamicin solution.

The first few days of the patient's postoperative course were largely unremarkable, as he was recovering well. On the fourth postoperative day, the patient began to have output from the colostomy. This is also when the pathology results from his appendiceal specimen indicated the patient had a well-differentiated NET measuring 0.13 cm and located 4 cm from the base of the appendix. No lymphovascular invasion was found. The tumor was not visible grossly by the pathologist. Serial dissections tested with immunohistochemical stains were positive for chromogranin and synaptophysin, indicating a NET was likely present.

Given the small size of the tumor and the lack of lymphovascular invasion, no further intervention was needed, as appendectomy is curative per the National Comprehensive Cancer Network (NCCN) guidelines [9]. The patient was discharged on the fifth postoperative day after tolerating food by mouth, achieving adequate pain control, and ambulating without assistance. Two days later, the patient presented to the emergency department with a large amount of frank red blood and pus oozing from the inferior portion of the incisional site. On physical examination, the midline abdominal incision was found to have seropurulent and persistent drainage. There was no erythema around the wound of the incision. The patient was afebrile but slightly tachycardic. However, he elected against medical advice to be discharged home, where he continued antibiotics and wound care.

**Figure 4:**
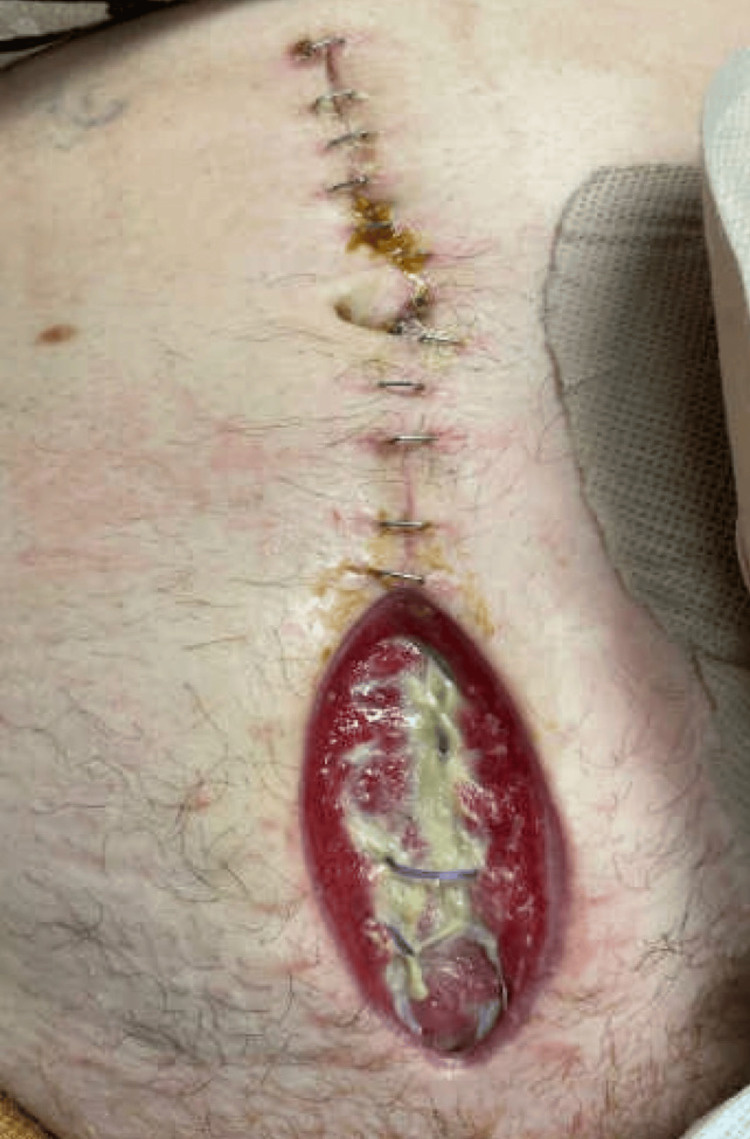
Midline abdominal incision with drainage

The patient presented to the emergency department the following day due to continued drainage of purulent fluid from the incision (Figure 4) and decreased colostomy output. He also reported compliance with wound dressing changes. The patient's white blood cell count was still elevated at 23,000 per microliter.

A CT scan with contrast found the patient had two intra-abdominal abscesses, one along the right hepatic lobe (measuring 9.9 cm at the largest point) and another in the left lower quadrant between small bowel loops (measuring 7.1 cm at the largest point). Interventional radiology was unable to obtain a safe window for intra-abdominal fluid collection. The lower left quadrant abscess was drained at the bedside with guidance from ultrasound. A culture swab of the abdominal wound indicated the presence of extended-spectrum beta-lactamase (ESBL) *Escherichia coli, *and the patient was started on ertapenem. Approximately one month after the finding of the intra-abdominal abscesses, a repeat CT scan showed a residual fluid collection that was significantly decreased in size. The patient's midline incision has healed well with no further signs of infection. The patient is scheduled for a colostomy reversal approximately three months after his original operation.

## Discussion

There is debate regarding whether the potential benefits of IA outweigh the possible drawbacks. IA can increase the risk of infection, lead to higher patient costs, prolong the length of surgery, and, subsequently, lead to an overall higher chance of surgical complications. Due to these potential downsides, the surgeon must perform a cost-benefit analysis that is individual to each patient. Some of the benefits associated with IA include eliminating the risk of the patient developing appendicitis and preventing metastasis of an appendiceal NET in an asymptomatic patient. IA tends to be more beneficial in patients already undergoing an exploratory laparotomy, women with a history of gynecological operations, and younger patients [10].

Initial management of rectal perforations includes IV fluid resuscitation, discontinuation of oral intake in preparation for emergent surgery, and broad-spectrum antibiotics. Diffuse peritonitis, as was seen in this patient, typically warrants an emergent exploratory laparotomy [11]. 

As seen in this case, most appendiceal NETs are incidental findings from appendectomies. The reported incidence of appendiceal NETs is 3-9 per 1000 appendectomies [2]. NETs are often located in the tip of the appendix, where they are unlikely to cause symptomatic obstruction. Conversely, approximately 10% of appendiceal NETs are located at the base, where they may cause an obstruction, resulting in appendicitis [2]. 

Appendiceal cancer is extremely rare. NETs are the most common type of appendiceal neoplasm, with mucinous adenocarcinoma being the second most common. Other types of appendiceal cancer include goblet cell adenocarcinoma, colonic-type adenocarcinoma, and signet ring cell adenocarcinoma [12].

Appendiceal cancers are classified as either epithelial or neuroendocrine neoplasms. Neuroendocrine neoplasms are further codified as well-differentiated NETs and graded from low to high based on the proliferation rate [2]. Approximately 70% of appendiceal NETs are considered well-differentiated, low-grade NETs [2], as was seen in the patient referenced in this case.

Similar to other NETs, appendiceal NETs can secrete vasoactive substances, such as serotonin, leading to the presentation of carcinoid syndrome. Carcinoid syndrome can present with right-sided heart valve disease, flushing, diarrhea, and wheezing. In 90% of cases, the progression to carcinoid syndrome represents metastasis (most commonly to the liver) [2]. 

The likelihood of metastasis for appendiceal NETs is generally determined by tumor size [2]. A comparative study from the University of Texas found that appendiceal NETs smaller than 2 cm were found to have nodal involvement in just 12% of cases [13]. The patient referenced in this case was asymptomatic with no metastasis beyond the 0.13 cm primary appendiceal tumor. This was confirmed by the specimen having negative margins and no lymphovascular invasion.

Patient prognosis is mainly dependent on the stage of the NET in question. NETs that have not metastasized and are isolated to the appendix have an excellent prognosis [2], as was seen in this case. 

According to the North American Neuroendocrine Tumor Society (NANETS) guidelines for managing and treating NETs, appendiceal NETs below 2 cm should be excised without additional intervention or follow-up [14]. Simple excision was the treatment regimen selected for the patient presented in the case above. Appendiceal NETs larger than 2 cm would warrant a recommendation of a right hemicolectomy with node dissection. These patients should have a follow-up appointment with a thorough history and physical, as well as imaging (CT or MRI) [14].

## Conclusions

Appendicular NETs may be suspected and detected during surgery and macroscopic examination of the appendix, and this finding may justify an appendectomy. As such, IAs in selective clinical scenarios where a risk-to-benefit analysis is favorable have the potential to reduce morbidity in asymptomatic patients with an appendiceal NET. 
